# Perioperative fluid management: Consensus statement from the enhanced recovery partnership

**DOI:** 10.1186/2047-0525-1-2

**Published:** 2012-06-27

**Authors:** Monty G Mythen, Michael Swart, Nigel Acheson, Robin Crawford, Kerri Jones, Martin Kuper, John S McGrath, Alan Horgan

**Affiliations:** 1 University College London. National Clinical Lead, Department of Health, London, UK; 2 Torbay Hospital, Torquay, Devon. Clinical Advisor in Anaesthesia, Department of Health, London, UK; 3 Peninsula Cancer Network, Consultant Gynaecological Oncologist, Royal Devon and Exeter NHS Foundation Trust, Exeter and joint National Clinical Advisor (Gynaecology), Department of Health, London, UK; 4 Addenbrookes NHS Trust. Joint National Clinical Advisor (Gynaecology), Department of Health, London, UK; 5 Consultant Anaesthetist & Associate Medical Director (Innovation & Improvement). National Clinical Advisor to Department of Health, London, UK; 6 Divisional Medical Director for Surgery, Cancer and Diagnostics, Whittington Health, National Clinical Advisor to Department of Health, London, UK; 7 Royal Devon and Exeter NHS Foundation Trust, Exeter and National Clinical Advisor (Urology), National Clinical Advisor to Department of Health, London, UK; 8 Newcastle NHS Trust. National Clinical Lead, Department of Health, London, UK

## Introduction

Enhanced Recovery (ER) after Surgery (or Fast Track) is a bundle of ‘best evidence based practices’ delivered by a multi-professional health care team, with the intention of helping patients recover faster after surgery [1]. Professor Henrik Kehlet, a surgeon from Denmark, pioneered the concept more than a decade ago but practitioners in the UK remained sceptical of his amazing results and adoption in the National Health Service (NHS) had been slow [1,2]. The Enhanced Recovery Partnership Programme (ERPP) was set up by the Department of Health in England in May 2009, to encourage the widespread adoption of ER with the aim of improving recovery from major surgery [1,3]. The Programme initially concentrated on elective major surgery in four specialities (Colorectal, Musculoskeletal, Gynaecology and Urology). Audit of ER practice by the early adopters demonstrated greater than 80% compliance with the majority of elements recommended by the ERPP. However, perioperative fluid management including the administration of pre-operative carbohydrate drinks and individualised goal directed fluid management guided by advanced haemodynamic monitoring (e.g. Oesophageal Doppler) had lower levels of compliance [3]. A pilot study using Commissioning for Quality and Innovation (CQUIN) to encourage practice change showed a dramatic improvement in outcomes in North Central London with very high levels of compliance with the ERPP recommended principles of perioperative fluid management, in particular goal directed fluid management [4].

The National Programme has evolved into the Enhanced Recovery Partnership (ERP), and the most recent guide published by the ERP includes evidence of widespread adoption of ER in the NHS in England and achievement of stated goals i.e. reduced length of hospital stay after surgery resulting in more operations being performed despite fewer bed days, no increase in readmissions and high levels of patient satisfaction [5]. Perioperative fluid management is at the heart of Enhanced Recovery and the use of intra-operative fluid management technology, such as Oesophageal Doppler, is supported by the ERP in line with the National Institute of Clinical Excellence (NICE) Guidance (MTG3), the NHS Operating Framework 2012–13 and the Department of Health Innovation Health and Wealth Review 2011 [5-7]. Despite concordance in the guidelines, the veracity of the evidence has been challenged [8,9].

The ERP thought it was timely to produce a consensus statement from the National Clinical Leads and Specialist Advisors within the specific context of Enhanced Recovery and, for the purpose of widespread dissemination, the general principles and key recommendations outlined in the latest guide are reiterated in this article [5]. Of note, no particular evidence based methodology was used aside from seeking unanimous agreement from the authors. A practical and pragmatic set of guidelines and recommendations was the aim. The conclusions do align with the GIFTASUP guidelines and NICE guidance where established EBM methodologies were utilised [6,8,10]. In making this consensus statement we agree that larger, more definitive studies of perioperative fluid management and, in particular, the relative contribution of haemodynamic monitoring compared with fluid restriction would be welcomed [11,12]. However, to be useful, such studies must be conducted in the context of a fully implemented Enhanced Recovery Program.

## General principles of enhanced recovery fluid management and recommendations of the enhanced recovery partnership

For further details see latest ERP guide [5].

### Pre-operative

· Maintain good pre-operative hydration.

· Give carbohydrate drinks.

· Avoid bowel preparation.

### Peri-operative

· Use fluid management technologies to deliver individualised goal directed fluid therapy.

· Avoid crystalloid excess (salt and water overload). ‘Maintenance’ fluid, if utilised, should be limited to less than 2 ml/kg/hr including any drug infusions. The use of isotonic balanced electrolyte solution (e.g. Hartmann’s) will minimise hyperchloraemic acidosis.

### Post-operative

· Avoid post-operative i.v. fluids when it is possible.

· Always ask the question; ‘what are we giving fluids for?:

Maintenance fluid?

Push early drinking and eating;

Replacement fluid?

Consider oral before i.v. and consider ‘prescribing’ oral fluids

Resuscitation fluids?

Use Goal Directed Fluid Therapy

**The Enhanced Recovery Partnership recommends the development of local guidelines and algorithms for fluid management and regular audit of compliance, in line with national guidelines, NICE recommendations and the Innovation, Health and Wealth Review (2011)**[6,8].

## Aims of ER fluid management (by the end of surgery)

· Patients core temperature is normal (circa 37°C).

· No evidence of hypovolaemia, tissue hypoperfusion or hypoxia.

· No evidence of hypervolaemia or excess fluid (‘zero balance’).

· Hb ≫ 7 g/dl.

· No clinically significant coagulopathy.

· Minimal use of vasopressors.

Predictors of poor outcome include: greater age, higher ASA status, high blood loss, longer than expected surgery, evidence of hypovolaemia or hypoperfusion (e.g. metabolic acidosis, blood lactate ≫ 2 mmol/litre, central venous O2 ≪ 70%), greater use of vasopressors, high volumes of i.v. fluids (≫ 3.5 litres total), positive fluid balance (≫ 2 litres positive on day of surgery) [13-18].

In an individual patient, failure to achieve these aims should prompt a review of the need for ongoing care in a higher care environment (e.g. extended recovery, HDU or ITU). If audit of a group of patients shows that these aims were not achieved, this suggests that the delivery of care should be reviewed.

## Individualized goal directed fluid therapy

**The Enhanced Recovery Partnership recommends the use of intra-operative fluid management technologies to enhance treatment with the aim of avoiding both hypovolaemia and fluid excess. This should be decided on a case-by-case basis adhering to local guidelines in the context of NICE recommendations, national guidelines and the Innovation, Health and Wealth Review**[6,8].

Indicators of central hypovolaemia include:

· Blood loss or fluid loss

· Tachycardia

· Hypotension

· Cool peripheries

· Low CVP

· Low cardiac output

· Reduced stroke volume

· Pulse pressure variation (during IPPV)

· Pre-load responsiveness

· Low central venous O2 saturation

· Raised blood lactate

Central hypovolaemia should respond to volume therapy (i.e. a fluid bolus) [13-18].

The Enhanced Recovery Partnership recommends that all Anaesthetists caring for patients undergoing intermediate or major surgery should have cardiac output measuring technologies immediately available and be trained to use them.

The use of intra-operative fluid management technologies are recommended from the outset in the following types of cases:

· Major surgery with a 30 day mortality rate of ≫ 1%.

· Major surgery with and anticipated blood loss of greater than 500 ml.

· Major intra-abdominal surgery.

· Intermediate surgery (30 day mortality ≫ 0.5%) in high risk patients (age ≫ 80 years, history of LVF, MI, CVA or peripheral arterial disease).

· Unexpected blood loss and/or fluid loss requiring ≫ 2 litres of fluid replacement.

· Patients with ongoing evidence of hypovolaemia and or tissue hypoperfusion (e.g. persistent lactic acidosis).

Perceived lack of resources is not a viable excuse. NICE have concluded that we can’t afford NOT to use intra-operative fluid management technologies where indicated [6]. Practitioners should not be constrained by lack of availability of such technology.

## What NICE said about cardio-Q Doppler

*“…The case for adopting the CardioQ-ODM in the NHS,…is supported by the evidence…The CardioQ-ODM should be considered for use in patients undergoing major or high-risk surgery or other surgical patients in whom a clinician would consider using invasive cardiovascular monitoring. This will include patients undergoing major or high-risk surgery or high-risk patients undergoing intermediate-risk surgery.”*[6]

## The GIFTASUP guidelines said

*“In patients undergoing some forms of orthopaedic and abdominal surgery, intraoperative treatment with intravenous fluid to achieve an optimal value of stroke volume should be used where possible as this may reduce postoperative complication rates and duration of hospital stay.”*[10]

## What the 2010 ER implementation guide: Delivering enhanced recovery said

“Individualised goal-directed fluid therapy…

When intravenous fluid is given, the benefits of maintaining circulatory filling and organ perfusion must be weighed against the risk of excess fluid accumulation in the lungs causing hypoxia, and, in the gut, causing nausea and delayed return of gut motility (ileus).

When there is not enough fluid in the bloodstream, the stroke volume falls – that is, there is a fall in the volume of blood ejected by the heart each heartbeat….

*In a typical regime enough colloid is given to maintain the stroke volume, but no more. This allows circulatory volume and organ perfusion to be maintained with the minimum of administered fluid, which minimises fluid accumulation in the tissues.”*[1]

The Enhanced Recovery Partnership recommends the regular audit of practice and outcomes benchmarked against national data for surgical procedures.

This should ideally include:

· 30 and 90 day mortality rate (ideally risk adjusted).

· Length of hospital stay.

· Same day admission rate.

· Readmission rate.

· Patient reported outcomes.

## Authors’ contributions

Each author contributed equally to this consensus statement. All authors read and approved the final manuscript.
